# A Critical Recuperation of Watsuji’s *Rinrigaku*

**DOI:** 10.1007/s11406-020-00296-1

**Published:** 2020-11-28

**Authors:** Aleardo Zanghellini, Mai Sato

**Affiliations:** 1 University of Reading, School of Law, Reading, UK; 2 Monash University, School of Law, Melbourne, Australia

**Keywords:** Watsuji, Ethics, Communitarianism, Coercion, Autonomy, Asian Ethics

## Abstract

Watsuji is recognised as one Japan’s foremost philosophers. His work on ethics, Rinrigaku, is cosmopolitan in engaging the Western philosophical tradition, and in presupposing an international audience. Yet Watsuji’s ethical thought is largely of niche interest outside Japan, and it is critiqued on the ground that it ratifies totalitarianism, demanding individuals’ unquestioning subordination to communal demands. We offer a reading of Rinrigaku that, in attempting to trace the text’s intention, disputes these arguments. We argue that Rinrigaku makes individual autonomy central to ethical action, despite the fact that its treatment of coercion may lead one to think otherwise; that it does not reduce ethical obligations to whatever demands any given society imposes on its members; that it draws a distinction between socio-ethical orders that are genuinely ethical and those that are not; and that, in insisting on the grounding of individuals in the Absolute, it makes adequate room for individuals’ resistance to unjustifiable socio-ethical demands.

## Introduction

In anticipating communitarianism and post-humanism, Watsuji’s work directly speaks to contemporary concerns not just in Anglo-American philosophical ethics, but the humanities more broadly. Yet Watsuji’s influence on Anglo-American ethical thought remains largely confined to Asianists, and even there the reception of his work has been mixed at best. Despite fairly recent sympathetic studies by McCarthy (2010, 2011) and (Japan-based academic) Sevilla (2017), and despite ‘a growing number of translations and full-length works testif[ying] to the rising awareness of Watsuji’s importance as a thinker in our problems today’ (Sevilla 2017, p. xxiii), the orthodoxy in Anglo-American academia is that Watsuji’s work suffers from considerable limitations.

As Odin (1992) summarises it, the main critique – both in Japan and the West – has argued that Watsuji’s communitarianism ‘has the tendency to slide into an impossible totalitarianism’ (p. 484), where ‘the individual is submerged in society’ (p. 491). More specifically, Bernier (2006, p. 89) argues that in Watsuji’s thought ‘the collective is fundamentally legitimate’; hence, ‘even resistance to a stiffened moral tradition appears in the final analysis as reprehensible’. Sakai (1991, p. 175) contends that, in arguing that ethics requires individuals to surrender to the call of totality, ‘Watsuji proposes … a kind of ethics that permits one to neglect other … ethical concerns in order to remain on good terms with others’. For Shields (2010, p. 270) it cannot be ruled out that by ‘call of the totality’ Watsuji means an individual’s capitulation to the ‘Emperor/State’. And Shuttleworth (2019, p. XX) argues that ‘Watsuji … has no concept of individual choice’, collapsing as he does the opposition between individuals and community until ‘there is no individual who can choose what it authentic, recognizing rather that one is one’s relations with others’. In this article we offer a critical but sympathetic reading of the first volume of Watsuji’s work on ethics, *Rinrigaku*, which disputes these arguments.

We are not the first to have defended Watsuji from such charges. Johnson (2016, p. 226) notes that Shields (2009) and Lafleur (1978) consider them inconsistent with Watsuji’s self-conception as a scholar ‘restoring a “middle way” between … atomistic individualism and … social organicism’, and that Kalmanson (2010) regards them as oblivious to the Buddhist ‘nondual metaphysics of dependent origination’ at the root of Watsuji’s thought. Our own strategy in defending *Rinrigaku* – specifically, its first volume – takes a different route. It relies on testing different possible readings against the text as a whole. We hope to show that, although some parts of *Rinrigaku* may invite the kind of hermeneutic moves made by Watsuji’s critics, the text as a whole ultimately remains recalcitrant to them.

In carrying out this critical recuperation of *Rinrigaku,* it is the text’s own intention (Eco 1994, pp. 44–63), rather than its author’s, that we will be after (even if we may often say ‘Watsuji argues…’ or ‘According to Watsuji…’). We follow Gadamer (1975) in thinking that authorial intention is ultimately irretrievable and, in any case, more of biographical than hermeneutic interest. Conversely, not only is a text’s intention (or at least what a text does not intend to say: Eco 1994, pp. 44–63) more amenable to being grasped; but sometimes readers are also better positioned than the text’s own author to appreciate the intention of a text.^1^

Because we are interested in the intention behind the first volume of *Rinrigaku*, we do not attempt to read the text in light of Watsuji’s other writings.^2^ Nor do we attempt to resolve interpretive questions in light of either the political pressures that, at different times, may have acted upon Watsuji’s working out of his account of ethics in the different volumes of *Rinrigaku* (see Sevilla 2014a, pp. 125–126), or the religio-cultural influences silently informing that account (see Nagami 1981; Lafleur 1978). Consequently, we also do not attempt a reading that balances both of these moves – that is, an inter-textual and a Buddhism-informed reading – at the same time (for such a reading see Sevilla 2017, pp. 175–221).

Our argument will be to the effect that *Rinrigaku* does not sanction totalitarianism, or demand individuals’ unquestioning subordination to communal demands (section 9). More specifically, we will argue that *Rinrigaku* does not reduce morality to whatever practices and beliefs any given society is committed to (section 2). This is because it draws a distinction between central (that is, ethically sound) and non-central cases of socio-ethical orders (section 5). It is also because it insists on the grounding of both socio-ethical orders and individuals in the Absolute (section 6), which gives individuals access to objective moral truths, enabling them to resist unjustifiable socio-ethical demands (sections 7 and 8). We will also argue that *Rinrigaku* makes individual autonomy central to ethical action (section 3), despite the fact that its treatment of coercion may lead one to think otherwise (section 4).

## Ethics as a Manner of Relational Life

In *Rinrigaku* Watsuji sets out to clarify the nature of ethics, a problem that he treats as inextricably linked to ontological questions about the nature of human beings. He describes *rinri* (ethics) as ‘a manner of the interconnection of acts that makes *ningen* [human beings] truly *ningen.* That is, it is a manner of *sonzai* of *ningen*’; where *sonzai* is the condition of existing/persisting in community (Watsuji 1996, pp. 21–22).

As rendered in the translation we are relying on (Yamamoto/Carter), this definition of ethics as ‘a manner of *sonzai*’ implies – via the use of the indefinite article ‘a’ – that human communities can subsist/persist even in non-ethical forms. That is, relational existence, which is characteristic of human beings, can take different forms: when it takes the form of *rinri* (namely, an ethical form), then it is constitutive of the central case of ‘human being’. In other words, being human, in the best and fullest sense of the word, means existing within human relations that are structured ethically rather than – as it is possible – otherwise. This reading rules out that Watsuji subscribes to the view that ethics can be reduced to whatever practices and beliefs a given society is conventionally committed to: it presupposes that something other than the mere fact of their social currency is required to qualify any concrete practices and beliefs as authentically ‘ethical’.

However, the Japanese language does not clearly distinguish between definite and indefinite articles. So, we cannot rest on the word choice of the Yamamoto/Carter’s translation our case for maintaining that Watsuji does not relativise ethics by reducing it to the concrete arrangements any given society happens to exist by. Rather, we need to show that, given the ambiguity in the original, Yamamoto/Carter render Watsuji’s definition of ethics correctly where they translate that ethics is ‘a’ (that is, one possible) manner of relational existence. We think that Yamamoto/Carter are indeed correct. We reach this conclusion through a *contextual* reading of the more ambiguous definition of ethics to be found in the original Japanese [「倫理とは人間を人間たらしめる行為的連関の仕方である。すなわち人間の存在の仕方である。」Our translation: ‘Ethics is a mode of interconnection of acts that makes humans human. Therefore, it is about how humans exist.’ We think this definition of ethics must be contextually understood in light of other passages of *Rinrigaku,* which are more explicit in ruling out that ethics can be reduced to the conventions any society contingently lives by. These passages will be discussed below. The conclusion we reach conflicts with the view that Watsuji’s communitarian ethics reflects ‘the general Japanese pattern of Japanese culture itself’ (Odin 1992, p. 492), whereby good and evil are conceived of as a function of ‘the group as the frame of reference, not as universal principles transcending the group’ (p. 493). Our reading will dispute that Watsuji’s ethical system lacks ‘any universalistic or transcendental standard relative to which individual and social action can be judged’ (Bellah 1965, p. 589).^3^

## Individuality

Watsuji (1996, p. 22) argues that human beings (*ningen*) have a ‘double structure’ – individual and social. More specifically, *Rinrigaku’s* central insight is that individuality is always contextualised within a web of human relationships (Frattolillo 2004): individuals and totality come to be established as the negation of each other, and human beings contain the double structure of individuality/totality.^4^

Let us turn to how Watsuji (1996, pp. 22–23) himself makes these points:

[T]he standpoint of an acting “individual” comes to be established only in some way as a negation of the totality of *ningen*. An individual who does not imply the meaning of negation, that is, an essentially self-sufficient individual, is nothing but an imaginative construction. … [But, similarly,] a totality that does not include the individual negatively is also nothing but a product of the imagination. These two negations constitute the dual character of a human being. … [W]hen an individual realizes herself through negation, a door is opened to the realization of totality through the negation of the individual. The individual’s acting is a movement of the restoration of totality itself.

Note Watsuji’s (1996) starting point: ‘the standpoint of an acting “individual”’ (p. 22). This emphasis on action is reinforced later: ‘The movement of absolute negativity is, first of all, a law of human beings… this law is closely tied to the practical and active spheres of human beings’ (p. 120). This clarifies Watsuji’s claim that the study of ethics should start with the study of *sonzai,* relational existence. An acting individual is a practical agent, that is, one who must decide, under the circumstances of relational existence, what to do. To be sure, we exist, and exist as practical agents, not just when we live in community; and not just when we are called upon to act in a moral way. But the case of a practical agent sifting through morally sound and unsound courses of conduct in the context of human relationships can be justifiably taken as the central (most salient) case of practical agency. Thus, Watsuji is right in saying that a self-sufficient individual is a construct of the imagination: such an individual would be one for whom the question of practical agency does not arise in its central form, that is in the form of how to conduct oneself morally towards other human beings.

Watsuji (1996, p. 23) clarifies what the movement of double-negation has to do with ethics:

[T]he true reality of an individual, as well as of totality, is ‘emptiness’ … [because] *ningen’s sonzai* is a movement of negation … [T]he basic principle of social ethics involves two [necessary] moments. One of this is the establishment of the individual as the other, over against totality. What is at stake here is the taking of a first step toward self-awareness. ... The other moment is the individual’s surrender to the totality … [T]he totality as community existence arises at the point where these many individuals become one by forsaking their individuality.

Sevilla (2014a, p. 112) notes that this account is somewhat puzzling: ‘But what does this self-awareness actually accomplish? Why take the detour towards individuality, if only to negate the individual again to realize totality?’ Sevilla (2014a, p. 105) believes that one should look to the third volume of *Rinrigaku* to resolve the ambiguities in the account of double negation contained in the first volume; if we do so, he argues, we can appreciate that ‘individuality … guides social change by intuiting how the totality ought to be.’ It is clear, then, that what troubles Sevilla (2014a) in *Rinrigaku’s* first volume is that, in insisting that individuality be negated in the interest of totality, Watsuji backgrounds the question of whether the demands of totality are justifiable.

Sevilla (2014a) expresses a legitimate concern; but we think we need not look so far as the third volume of *Rinrigaku* to address it. Specifically, we think that, in arguing that ethics demands that the individual, having negated totality, be negated and return to it, *Rinrigaku* has in mind not just any society, but the case of *ningen sonzai* (relational existence) in which *ningen* (humans) are truly *ningen*. This, as we already know, is the case in which relational existence is structured ethically: that is, the case in which societal obligations (the demands of totality) are morally justified. If we follow the text in making this implicit assumption, then it becomes apparent both why individuals, having negated totality, ought to return to it, and why they ought to have negated it in the first place. They ought to return to totality because, by definition, doing so means meeting one’s ethical obligations, and obligations are precisely that – ought-statements. Yet, before a practical agent’s compliance with (justifiable) societal demands can be said to be truly ethical, she must have gained awareness of herself as an *individual capable of moral choice*. Staying within totality is not enough: If I am to act truly ethically, I ought to negate totality’s demands (that is question their validity, at least hypothetically), and then I ought to return to totality. In fact, as we will see below, it is precisely in this process of double-negation that the individual becomes capable of assessing whether societal demands are morally justifiable (as they are in the central, that is the ethical, case of *ningen sonzai*), and therefore whether a surrender to totality is called for.

That is, in the passage just quoted Watsuji is telling us that ethics (the central case of practical agency) requires, in the first place, individuals to be aware of themselves as practical agents who *could* choose to fail to comply with the (legitimate) demands of community. Then, in the second place, ethics requires them to restore relational existence by choosing to do the right thing, namely, complying with those demands (which are by hypothesis justifiable in the context of the central case of *ningen sonzai*).

In short, ethics demands autonomy: sound ethical prescriptions must be complied with autonomously, rather than heteronomously (Kant 2009, pp. 50–51). We use this Kantian terminology loosely, to stress that one’s performance of ethical duty requires (at least in most cases) that the reason for it be one’s recognition of it as good, rather than some extraneous (heteronomous) considerations related to the source of the prescription enjoining the duty (God, society, convention, a figure of authority, State law, etc). As Raz (1984, p. 141) explains: ‘We expect people to avoid … [misdeeds such as rape and murder] whether or not they are legally forbidden, and for reasons which have nothing to do with the law. If it turns out that those reasons fail, that it is only respect for the law that which restrains them from such acts, then those people lose much of our respect.’ These observations about legal prescriptions can be extended to social prescriptions.

We think, therefore, that in stressing that ethics requires not just staying within community, but gaining consciousness of individuality before returning to community via the performance of social obligations, *Rinrigaku* rightly makes individuals’ moral autonomy central to ethics. Consciousness of individuality enables us to meet a socio-ethical obligation not unreflectively, just because it is the done thing, but because we autonomously appreciate and will it as good. The practical consequences of our action may be the same regardless of the reasons we may have for complying with socio-ethical obligations; but we are not truly acting ethically until we gain consciousness of ourselves as individuals: free, self-determining agents capable of moral choice. On this view, the surrender to totality that ethics requires is the autonomous endorsement of moral norms emerging out of the central case of *ningen sonzai*, i.e. genuinely ethical relational existence. To forsake individuality in the interest of realising totality here is to subordinate self-interest (prudence) to the imperatives of (sound) morality.

## Coercion and obligation

The main challenge to this reading of *Rinrigaku* comes from its treatment of coercion. As we know, human beings have a negative structure. In this scheme, coercion suppresses individuality in the interest of the whole. Coercion, specifically, effects ‘the negation of the standpoint of the individual’, and to that extent coercion indeed ‘is involved’ in the ‘socio-ethical unity’ that results from individuals ‘return[ing] to themselves through the negation of negation’ (Watsuji 1996, p. 139).

Watsuji (1996, p. 85) endorses Hobbes’ view that ‘coercion is required to ensure that an individual will obey the laws of society’, because humans naturally ‘rebel against communal life’. To show that socio-ethical wholes have coercive power, which motivates individuals’ compliance with socio-ethical norms, Watsuji (1996, p. 108) uses the example of a community of love:

love affairs demand exclusive possession … [and this] restricts individuals through strong coercive power. Therefore, the increase of universalisation and the strengthening of coercion cannot be separated from one another … We cannot speak of universalization (and hence, of social forms) without having recourse to the negation of the individual. (Watsuji 1996, p. 108).

It is apparent that Watsuji here uses ‘coercion’ in a relatively loose sense – one that he inherited from Durkheim. Coercion, for Durkheim (2013, pp. 20–23), is characteristic of all social facts – namely, ways of thinking, acting, and feeling (including those prescribed by laws, customs and morality) that are generalised throughout a society, exceed the lifespan of any one individual, and impose themselves on individuals quite regardless of their will. Durkheim equivocates between the idea of coercion properly-so-called (which implies the use of *force* and/or *lack* of options) and the looser idea of ‘constraint’ (Durkheim 2013, p. 23). In doing so, he assimilates social penalties (such as criticism or ostracism) that attend the breach of moral duties, with genuine coercive effects, such as legal sanctions, or the compulsory use of a country’s language or currency if one wants to speak or transact there.

To be sure, there is nothing that precludes either Durkheim or Watsuji from assigning ‘coercion’, by implicit stipulation, such an expanded meaning. But Watsuji also follows Durkheim (20,134, p. 22) on a more problematic claim: namely, that because ‘most of our ideas and tendencies … come to us from outside, they can only penetrate us by *imposing* themselves upon us’ (emphasis added). This is a non-sequitur, for I can embrace an idea or moral precept originating outside me, without any form of imposition. Even if it may be true that much of my ideas were more or less ‘imposed’ on me through education in childhood (Durkheim 2013, p. 23), insisting on the idea of ‘imposition’ obscures the fact that Socratic self-examination is not thereby precluded to me. Durkheim himself recognises this in his later work on moral education (see Smith 2014, p. 23, 26, 28).

Watsuji’s treatment of coercion reflects some of these ambiguities in Durkheim’s work. This, we think, explains why in the passage quoted above, about the coercive power of love affairs, Watsuji (1996, p. 108) establishes an equivalence between coercion and the negation of individuality that results in norm-compliance. Indeed, later Watsuji (1996, p. 139) performs a wholesale rhetorical conflation of obligation and coercion: ‘[C]oercion constitutes the essence of obligation. … [O]bligation requires self-negation with an imperative authority, and the thus negated individual obeys this authority out of respect’ (p. 139).^5^

However, Watsuji must have known – as Durkheim himself realised (Smith 2014, p. 23, 26, 28) – that individuals do not comply with communal demands only when they are made to (via coercion), or out of fear of sanctions (that, is because of the *threat* of coercion): just as often they comply because they think it is the right thing to do. The very example of love affairs, used by Watsuji to illustrate the centrality of coercion in realising totality, actually proves that coercion is merely incidental to this realisation. For one could not truly participate in the distinctive good that is a monogamous love affair, if the only or main reason for one’s fidelity to one’s lover were a heteronomous one based in coercion or the threat thereof. Thus, we think that *Rinrigaku’s* points about coercion – if we are to make good sense out of them – must be understood to mean that coercion is required *only to the extent that individuals fail autonomously to return to community (that is, willingly and non-heteronomously to comply with community’s legitimate demands)*. If one is to salvage the ethical picture drawn by *Rinrigaku,* then this implicit qualification must be built into its account of coercion.

What led Watsuji astray here was his determination to conceptualise coercion and individuality as co-implying each other. We already know that for Watsuji we are individuals only insofar as we either eschew, or contemplate eschewing,^6^ the demands of socio-ethical obligation. Watsuji’s treatment of coercion makes the mistake of thinking that when we voluntarily comply with such obligations, we lose consciousness of our individuality – a consciousness that, on the other hand, coercion preserves. Watsuji (1996, p. 114) illustrates this by analogy:

people who engage in a communal rope pull with sincerity combine to become one subject. It is not that I pull and that you also pull but that “one power” somehow pulls. On the other hand, “coercion” consists of forcing separated individual subjects to subordinate themselves to the whole.

So, for Watsuji (1996, p. 116) individual revolt and societal coercion are the forcible/violent movements that co-imply each other and that explain the negative structure of human beings: only when I am *made* to obey do I realise totality *without losing my individuality* (this is because coercion, being involuntary, does not affect consciousness of my independence).^7^ This point, too, smacks a little of Durkheim – particularly his idea that ‘conforming to the normal order of things because one is … sure … that everything is as it ought to be’ does not qualify as a genuine moral act, for that kind of conformity does not involve ‘submitting to a constraint’ (Smith 2014, p.28). Watsuji may have been misled by this point into thinking that it is only if I feel compelled against my will that I can experience myself as an individual (a practical agent capable of autonomous choice); hence only under such coercive circumstances does my conformity have a moral quality.

To take stock: in *Rinrigaku’s* account, individuality consists in the consciousness of one’s self-interest as in opposition to socio-ethical obligations; only through ‘revolting against the whole … does the consciousness of the ego come to be established’ (Watsuji 1996, p. 134). One’s performance of obligation can take two forms under this scheme: either one complies because one is made to, coerced, so that one’s compliance is not truly consensual, and it is in that tension that one’s consciousness of individuality is maintained; or one complies because one sincerely wants to (as in the rope pull example), but then it is not really one who is acting, but the whole, in which one has already lost one’s individuality. It is apparent that this conceptual economy makes difficult to accommodate precisely the individual’s non-heteronomous endorsement of socio-ethical obligation that we have discussed in the previous section.

Yet, pace *Rinrigaku’s* treatment of coercion, the central case of negation of individuality should be seen precisely as the individual *autonomously* deciding to subordinate her self-interest to the common good, either because this is a requirement of justice (in which case the individual negates unjustifiable individualistic impulses or desires), or as a matter of supererogation, that is, as a way of going beyond the call of duty (in which case the individual willingly negates her self-interest despite the fact that society cannot rightly demand it of her). Coercion only comes into play when autonomous performance of duty does not.

There are, happily, moments in which *Rinrigaku* explicitly recognises precisely something along these lines, abandoning its view that individuality and coercion co-imply each other. This is especially apparent where Watsuji (1996, p. 138) shows how the concept of freedom is nothing other than ‘the fundamental law of a human being’ described by the movement of negation. On the one hand, the freedom-from that preoccupies liberal political philosophers describes the first movement of negation, which consists in an individual emancipating herself from society; on the other hand, the idea of freedom as self-causation, as autonomous self-determination à la Kant (‘according to Kant’s explanation, this autonomy consists of the control of the individual self by the superindividual and authentic self’) describes the return to the whole.

This passage cannot be reconciled with a view that takes at face value Watsuji’s claims about the co-implication of coercion and individuality. Rather, it implicitly qualifies those claims, making room for a return totality that proceeds from one’s moral autonomy rather than from coercion. Indeed, in admitting that the autonomous performance of moral duty qualifies as the negation of negation that restores the totality of *ningen* in the distinctly ethical way – that is, by retaining *ningen’s* double structure – this passage implicitly denies what Watsuji (1996, p. 114) had tried to establish with the rope pull example: namely, that voluntariness in the performance of duty results in the dissolution of one’s individuality into the whole. It is with good reason, that Watsuji (1996, 138) now implicitly recants that point. While pulling a rope with sincerity, I may know very well, for example, that I will choose to assert my individual will against peer pressure and choose not to partake in the next game; by the same token, when I autonomously perform a socio-ethical obligation, the only thing that dissolves is my resistance to the performance of that particular obligation, but that resistance is not all that the consciousness of my individuality consists in. In short, in the passage where it discusses freedom-from and freedom as self-causation (Watsuji 1996, p. 138), *Rinrigaku* implicitly concedes that consciousness of individuality fails us no more when we autonomously perform socio-ethical obligations than when we are coerced into them.^8^

Likewise, Watsuji’s (1996, p. 135) critique of the crowd instinct is clearly predicated upon the possibility of a kind of conformity with norms that is neither coerced, nor like his example of the rope pull (where individuality, supposedly, disappears). This kind of norm-conformity results from individuals *autonomously* choosing to be community-oriented and to comply with socio-ethical obligations when confronted with the possibility to revolt against such obligations out of egotism. Specifically, Watsuji (1996, p. 135) is clear that unthinking or mechanical conformity to socio-ethical demands is not praiseworthy: although it makes ‘existence of society as an organic whole’ arise, it is at the cost of individuals losing their ‘self-conscious essence’ and spending ‘hours in idle slumber in a community, falling victim to the “crowd”’. The logical alternative to such forms of social conformity is, clearly, just that non-heteronomous performance of socio-ethical duty that elsewhere Watsuji’s preoccupation with coercion tends to obscure.

## Between Conventionalism and Objectivism

In the previous sections we have already sketched the thrust of our argument about how *Rinrigaku* should be read: the movement of double negation draws attention to the fact that personal autonomy is key to genuinely ethical action. It is not just that awareness of one’s individuality ‘is a paradigmatically ethical moment, insofar as a weak sense of self as individual puts one in danger of everything from falling into the mindless conventionality of the crowd to developing a willingness to submit to powerful external forces’ (Johnson 2016, p. 226). Rather, even when the demands of totality are morally justifiable, a weak sense of self partly evacuates the performance of ethical obligations of its ethical value: for conduct to be truly ethical, we must gain awareness of ourselves as self-determining subjects, even if only to then put our self-determination in the service of community-oriented social obligations that codify sound ethical imperatives.

This reading is contingent on assuming that wherever *Rinrigaku* states that ethics means surrendering to the call of totality, it is assuming a surrender to the central case of *ningen sonzai,* that is, a community whose demands on its members are morally sound. Is that assumption justified, though? Or does *Rinrigaku’s* account, rather, end up automatically ratifying as ethical everything that any given community may happen to demand of its members at any particular time (as long as individuals autonomously endorse those demands)?

The question arises because there are many ambiguous statements in *Rinrigaku,* which are compatible with either view on ethics. We have already drawn attention to a passage (Watsuji 1996, p. 22) that – depending on how one construes the ambiguity generated by the lack of distinction between definite and indefinite articles in Japanese – can be read as either implicitly affirming or denying that communal life can ever take unethical forms. Similarly, several other passages are compatible with either an objectivist or a conventionalist conception of ethics.

Take the following: ‘If I perform some obligation I am not actually obedient to an obligation that I myself set forth but to the social order I have inherited from being taught, and such order is thought to be objectively grounded in law and morality’ (Watsuji 1996, p. 111). The expression ‘objectively grounded in … morality’ may suggest an objectivist stance on morality, which sees the latter as a matter of moral facts or truths. But Watsuji’s bringing law into it – given that we are all familiar with the existence of bad laws – muddles the waters. It may suggest, specifically, that morality here is treated as objective only in the same way in which law is. That is, what the word ‘objectively’ might imply is that morality (like law) can be objectively ascertained by looking at a community’s *social* practices and/or beliefs (this is a conventionalist understanding of ethics); not that morality is an objective fact potentially in conflict with those practices and beliefs (this is an objectivist understanding of ethics).

Likewise, Watsuji’s (1996, p. 134) point that goodness ‘may also be achieved by recognizing another community’, rather than by going back to the socio-ethical whole one has revolted against, could also be interpreted in either a conventionalist or objectivist sense. The conventionalist interpretation is that the content of one’s obligations (as determined by different communities’ practices) does not matter, as long as one obeys *a* community’s obligations. The objectivist interpretation is that revolt against one’s socio-ethical order may lead us towards other, more genuinely ethical, socio-ethical wholes (compare Sevilla 2014a, p. 113).

When *Rinrigaku’s* discussion starts to foreground the Absolute, however, these interpretive ambiguities can be dispelled. We already know that it only makes sense to speak of ethics where revolt against the socio-ethical whole (or totality) is either contemplated or enacted; and where conformity with duty occurs by way of further negation (Watsuji 1996, p. 121) – either through (threat of) coercion that motivates compliance, or the autonomous endorsement of socio-ethical obligations. Because what makes *ningen* truly *ningen* is relational existence structured by ethical norms (Watsuji 1996, p. 22), and because ethics is the movement of double negation, ‘the true reality of an individual, as well as of totality, is “emptiness”’ (p. 23), that is, ‘absolute negativity’, which Watsuji says is the ‘fundamental source’ of individuals (p. 117).^9^ For both individuals and the concrete socio-ethical wholes to emerge as finite, individuated entities out of the Absolute/absolute negativity, their ultimate essence as emptiness or absolute negativity must be negated (Watsuji 1996, p. 120). In practice, however, it is by opposing *each other* – rather than, strictly speaking, the Absolute/absolute negativity itself – that individuals and socio-ethical wholes establish their being^10^:

The negation of absolute negativity [that] establishes the standpoint of an individual … always takes place in the form of a revolt against the whole *as something socio-ethical* … Similarly, the standpoint of the whole is established as the negation of negation of absolute negativity ... But this should not be taken to signify a mystical experience in which the individual is immersed in the Absolute. The sublation ... of the individual’s independence that arises as the negation of negation occurs *in the form of its subordination to the socio-ethical whole* … which appears in various forms, such as family, friends, a company, a state, and so forth. ... [R]egarding this union of an individual with the whole, mention can be made of a superindividual will, or the total will, or of obligatory acts and so forth (Watsuji, 196, pp. 120-121, emphasis added).

Of course, there is nothing that guarantees that finite socio-ethical wholes individuate themselves out of the Absolute in ethically sound forms: socio-ethical obligations, as defined by the finite socio-ethical whole’s superindividual will, may or may not impose justifiable demands on individuals. Yet participation of individuals in the Absolute, and hence in authentic justice, can only occur via their relationship with the finite socio-ethical wholes against which they revolt, and to which they return. That is, concrete, finite socio-ethical orders are the places where justice is enacted and aspired to. Authentic justice will not be fully realisable within these orders, given their finitude; and yet they are the only places where striving towards authentic justice can occur. As Watsuji (1996, p. 122) explains, universal brotherhood is ‘“an ideal”, not a socio-ethical reality’, and yet this does not make it ‘simply nonsense.‘ If we were to try to realise it, ‘from where else should we start other than from just now? Unless we recognize that the finite socio-ethical whole is precisely the place of the self-return of the Absolute, then this ideal would, in the final analysis, fall victim to the charge of meaninglessness’.

So, in appealing to the Absolute, Watsuji’s account conjures up a disjunction between conventional morals (a concrete community’s practices and or beliefs) and the demands of authentic justice (the standard against which those practices and beliefs are to be judged). At the same time he insists that it is only within concrete socio-ethical wholes that the aspiration towards justice can be worked out and (partially) realised.

This point comes across particularly clearly when Watsuji (1996, pp. 122–123) discusses Buddha and Christ, who, in revolting against the socio-ethical whole, managed what is not possible for the rest of us, that is, to stand in a genuinely direct relationship to the Absolute (as opposed to experiencing it as mediated by the socio-ethical forms of the community). Yet, as Watsuji is keen to point out, these figures later went back to their community to realise moral precepts within the finite socio-ethical whole.

Here Watsuji’s account draws a distinction between socio-ethical orders as they are and as they ought to be. The problem is that, unlike Buddha and Christ, we cannot judge socio-ethical wholes by standing outside them, in a position that gives us direct, unmediated access to the Absolute and authentic justice. A society in which ‘each individual is tied to the Absolute’ – Watsuji (1996: p.121) calls it an ‘open society’ – is an abstraction: it would not be a human society in the first place, it would be something that subsists in a form other than *sonzai*. Yet, despite this lack of direct access to the Absolute, *Rinrigaku* wants to make room for our ability to grasp ethics in a way other than one based purely on conventionalism.

This ability is clearly presupposed where Watsuji (1996, p. 189) speaks of individuals discarding socio-ethical wholes only to find that the authentic socio-ethical whole is thereby foregrounded. An even clearer stance against pure conventionalism is apparent where he discusses closed societies. These are just the communities or socio-ethical wholes we are all familiar with: they are characterised by non-universal (i.e. exclusive) membership,^11^ and by the fact that they demand conformity to their practices/conventions/laws (Watsuji, 121–123). Crucially, Watsuji (1996, p. 123) warns us against treating our communities, that is closed societies, as ‘an ultimate aim’:

A family or a state, as instances of socio-ethical wholes, has the authority to demand obedience from individuals, but this demand cannot be authorized merely from the standpoint of a family or a state. Where an individual who revolted against a family or a state, finds himself based in the Absolute, then by what right can a family or a state, as finite wholes, demand the negation of this individual? Even the prosperity of a state, insofar as this state is but a finite group of human beings, is not given priority over the dignity of an individual who originates in the Absolute.

We do not think there is a way of making sense of this passage that can dispense with the idea that the authority of socio-ethical wholes ultimately depends on the extent to which their demands are objectively justifiable. If our complying with their authoritative directives is likely to improve conformity with reasons that apply to us (reasons being facts about what is of value in the world), then those directives have legitimate authority over us, but not otherwise (Raz 2006).^12^ The question then is how we can make reliable judgements about situations in which non-compliance with societal demands is called for, because those demands are not justifiable in ethical (or other) terms. Though we cannot have direct access to the Absolute, we are ‘based’ and ‘originate’ in it (Watsuji 1996, p. 23), and it seems that it is precisely the movement of negation – when we take it upon ourselves to question our finite socio-ethical wholes, as a prelude to returning to them – that activates that origin or grounding in the Absolute, affording us insight into objective values, including moral facts, and enabling reasoned resistance against societal demands that violate those moral facts.

*Rinrigaku’s* treatment of evil, too, suggests its allegiance to a non-conventionalist understanding of ethics. Its description of ‘badness’ as the ‘movement’ of ‘rebellion’ against ‘one community or another’ (Watsuji 1996, p. 133) might lead one to think otherwise; but, as an illustration, Watsuji (1996, pp. 133–134) states that in all religions as well as, before them, in ‘tribal societies’, ‘the separation from the whole due to hatred or egoism has been proclaimed “evil”’. The qualification ‘due to hatred or egoism’ appeals to objective standards that would have been omitted if it were merely the fact of revolt against the whole in and of itself that were crucial to something qualifying as evil. The same is true of the qualifier ‘authentic’, in the statement that the ‘standpoint of the “self” is that of the opposition between self and other that has departed from authentic wholeness’ (Watsuji 1996, p. 189).

## Knowing Moral Truth

We have argued that, although we cannot have direct access to the Absolute, we are always grounded in it, and it is precisely the movement of negation – the point at which one questions one’s socio-ethical obligations – that affords us insight into moral facts. Watsuji’s discussion of the Kantian requirement of norm universalizability confirms this reading.

The judgement that one’s action, as mandated by a socio-ethical obligation, conforms with a universalisable prescription, out of which ‘the imperative to act arises’, implies a ‘superdiscriminative, authentic self as noumenon’: this is a ‘supraindividual rational will that lies at the rear of the individual will’ that has revolted against the obligation (Watsuji 1996, p. 139). That finite, individual will has emerged precisely ‘in separation from [that] superindividual, superdiscriminative foundation’; as a result of that separation, ‘congruence with the universal is again demanded’ through an individual’s return to ‘the superdiscriminative as its ground’ (p. 139). We have seen, however, that, according to *Rinrigaku*, participation of the individual in the Absolute, and hence in the superdiscriminative, can only occur via their relationship with the finite socio-ethical whole. So, genuinely ethical actions occur – where they do – precisely in the form of a movement of revolt against (in the sense of questioning of) and a (discerning) return to the demands of the finite socio-ethical whole. More concretely, access to moral truth requires critical engagement with a communal context and its concrete prescriptions. This makes intuitive sense, if only because of the communal character of the good of knowledge (including knowledge of moral facts).

Watsuji (1996, p. 245) offers examples of this dependence of objective moral judgements on social forms: ‘Whereas Kant took for granted the presupposition that not to gain excessive profits is to act in accordance with one’s obligation or that one is obliged to sell goods honestly, nevertheless, the problem of what constitutes excessive profits or honesty can be resolved only socially’. Kant’s examples of the duty to preserve one’s life and to show benevolence towards the less fortunate are also found, by Watsuji (1996, p. 246), to be not self-standing (as Kant implies), but dependent on the social: ‘In one’s being, one has a position that is determined by various forms of the whole’ and it is due to this socially determined position that the obligation to preserve one’s life ‘matter[s] at all to ethics’. Likewise, ‘benevolence’ is a duty only in societies where inequalities of power and wealth exist: where equal status is enjoyed, one would ‘think it shameful to be shown mercy’ (p. 247).

Note that these passages do not propose a conventionalist account of morality, whereby the badness or goodness of something is reducible to a function of the conventions of any given socio-ethical whole. It is no doubt true that honest selling and excessive profits only make sense in the context of human practices such as business, and that these practices, and the prescriptions that go with them, vary (Watsuji 1996, p. 246). But the requirements of honesty or avoidance of greed in business are based on more general principles/virtues that exceed the business context and thereby manifest themselves in social spheres different from business (indeed, they would manifest themselves even in societies where business did not exist). As to the duty to show benevolence and the duty to preserve one’s life, it might or might not be right that they are socially contingent in the way *Rinrigaku* claims they are. But, either way, the good of life or value of sympathy are not themselves so contingent; so, a shift in communal practices would not change the objectivity of those values, thought it might change the content and significance of the duties derived from them. So, the dependence of moral judgements on the particular social forms of finite and concrete socio-ethical wholes does not mean that moral facts themselves are also so dependent. To put it differently, it is not justifiable to draw, from *Rinrigaku’s* emphasis on the contingency of the validity of specific socio-ethical obligations, the conclusion that the values that underlie those obligations (moral facts) are themselves contingent.

What *Rinrigaku* does insist on, is that we only come to know of those moral facts by interrogating the concrete socio-ethical obligations associated with them in the context of the social forms of finite socio-ethical wholes, not by harnessing anything like pure reason. Watsuji (1996, p. 252) is clear on this: he argues that Kant is right in taking as determinative of what counts as genuinely ethical conduct the congruence between ‘the subjective principle determining the individual will (i.e., the maxim) and the objective one (i.e. the moral law)’ – for after all ‘[t]his correspondence is what we had in mind by means of what I term the *individual*/*total* basic structure of *ningen sonzai’*. Nonetheless, he notes, the moral law itself is not grounded purely in logic or pure reason, almost as if the requirement of universalisation, coupled with appeal to some purportedly a-priori considerations, is enough to allow us to determine on the permissibility/obligatoriness of a course of conduct. Rather, the moral law is something we can only figure out ‘from the standpoint of human relationships’, from ‘the spatio-temporal dynamic structure if *ningen sonzai*’ (Watsuji 1996, p. 250). There are, here, echoes of Rawls (1971, p. 587), where he describes what he calls the ‘standpoint of eternity’ as ‘not a perspective from a certain place beyond the world, nor the point of view of a transcendent being’, but ‘a form of thought and feeling that rational persons can adopt within the world’. But Watsuji emphasises the communal nature of that world, reminding us that the enablijg condition for the very rationality of rational persons is the self’s embeddedness in the socio-ethical whole.

## An Objectivist Perspective on Moral Facts

We argued that although socio-ethical obligations and their validity are contingent on the social forms of finite socio-ethical wholes, the moral facts on which they are based are not. *Rinrigaku* is explicit about this when discussing trust and truth(fulness). Watsuji (1996, pp. 265–282) argues these are necessary to *ningen sonzai:* authentic wholeness requires and presupposes trust and truth, and so do the concrete communities that do more or less of a good job of instantiating authentic wholeness. Indeed, ‘human relationships are those of trust’ (Watsuji 1996, p. 271) and ‘at a place where truthfulness does not occur at all, *ningen sonzai* perishes … [T]he fixation of evil, radical evil, consists precisely in preventing the truthfulness of *ningen sonzai* from occurring*’* (p. 282).

Like his discussion of the Absolute, Watsuji’s remarks about truthfulness and trust are an important counterpoint to the passages in *Rinrigaku* that could be interpreted as supporting a conventionalist stance on morality. In fact, not only truthfulness and trust, but also certain duties grounded in them are inseparable from *ningen sonzai*, and as such could be said to qualify as objective moral facts applicable across time and space. Watsuji (1996, p. 287) mentions the prohibitions against homicide, adultery, theft and deceit, shared by the Five Commandments of Buddhism and the Ten Commandments of Christianity. These acts involve a betrayal of trust and hence a revolt against truthfulness: as such their wrongness can be determined ‘on the basis of *ningen sonzai* and therefore remains constant, in any time or place’ (p. 287). This is perhaps the single most objectivist statement in *Rinrigaku.*

Yet*,* the import of this statement is almost negated by the way in which Watsuji responds to the objection that different societies have widely different practices in respect of homicide, adultery, theft and deceit. Watsuji concedes this, but explains that the prohibitions remain constant: it is their scope of application that varies depending on how encompassing the relationships of trust are in any given society. So, homicide is always considered wrong, but killing an enemy or the sacrifice of a designated victim do not count as homicide in societies that condone these practices because there is no relationship of trust between the victim and the executioner, and hence no shared expectation that the duty will be complied with in relation to victims. Similarly, if in heroic societies the powerful are allowed to plunder, that is because plunder does not really count as theft, as the weak do not trust the powerful will abstain from appropriating the spoils of those they defeat. On the other hand, Christianity preaches universal brotherhood, and extends trust to all human beings, thus making all human killing count as homicide; and Buddhism extends trust to all living things, so animal killing counts as homicide, too (Watsuji 1996, pp. 88–291). Watsuji (1996, p. 291) concludes that, therefore, ‘the view that the standard of goodness and badness differs in accordance with time and place is obviously false. What differs here has something to do with the extent of the trust relationship and the manner of its expression, not with the principle according to which a response of trust is good and a betrayal of trust bad’.

A moral objectivist reading this passage might well conclude that *Rinrigaku’s* own moral objectivism is hollow, if, together with the (constant) moral wrongness of theft, homicide, etc., it accepts at face value the ways in which different communities have historically confined and continue to confine the benefits of prohibitions against theft and homicide on morally arbitrary grounds. Sakai (1991, pp. 183–185) is right, here, in critiquing the conception of trust that seems to emerge from passages of *Rinrigaku* such as this. As he argues, sociality is not just the ability to ‘operate within pre-arranged social relations, such as parent-child and employer-employee. Sociality … mean[s] the ability to leave behind the sort of trust warranted by the already existing relations, to “go out into the world” and to establish new relations with strangers’ (Sakai 1991, p. 184).

Yet we should not necessarily assume Watsuji would disagree. In discussing theft, homicide, adultery and deceit, Watsuji may not expressly point out that relationships of trust have been historically confined by many societies on morally arbitrary grounds. But just because he does not say so, does not mean he does not believe it. After all, recall his statement that if universal brotherhood is ‘an ideal’, it is not thereby ‘simply nonsense‘(Watsuji 1996, p. 122).

## The Call of Totality

We are now in a position to address the problem we started with: does *Rinrigaku* ratify an ethical vision in which individuals are wholly subordinated to the totality, in the contingent forms in which any given socio-ethical whole instantiates it? Consider statements such as: ‘The supreme value is absolute wholeness; and an aspiration, or upward drive, or eagerness for it, is “goodness”’ (Watsuji 1996, p. 134); and ‘Conscience is the call of the original totality; … good and evil consist respectively in going back into and going against the direction of this movement’ (p. 23). Do not these passages betray a kind of communitarian ethics where the individual and her rights count for precisely nothing?

We think *Rinrigaku* does not authorise us to draw such conclusions from these statements. For all that we can only experience the Absolute in the context of concrete socio-ethical wholes, *absolute* wholeness and *original* totality are clearly attributes of the Absolute, not of concrete socio-ethical wholes. Thus, re-establishing absolute wholeness or heeding the call of the original totality may or may not involve subordination to the demands of the concrete socio-ethical whole against which we revolted – it depends on how those demands measure up. In light of Watsuji’s (1996, p. 123) argument about the dignity of individuals grounded in the Absolute, *Rinrigaku’s* moral objectivism, and the room it makes for our knowledge of moral truth, we think it is an interpretive mistake to read passages such as these as requiring individuals to make endless concessions to the community. Rather, *Rinrigaku* is consistent with the view that wholeness is genuinely realised only where everyone is given their due, and when acts of self-sacrifice that go beyond the call of duty are recognised as supererogatory, rather than obligatory.

## Conclusions

We have proposed a critical recuperation of *Rinrigaku* in response to arguments that *Watsuji’s* communitarian ethics conflates the ‘is’ and ‘ought’ of morality, and that it has a troubling anti-individualistic bias. The key original insight of *Rinrigaku* is that ethics is a movement of double-negation. When properly understood, this insight makes individual moral agency central to ethical action, by insisting that to truly qualify as ethical, compliance with a socio-ethical obligation requires the agent to non-heteronomously endorse the obligation. Coming to this conclusion requires reading the parts of *Rinrigaku* that deal with coercion against the grain – but there is enough in the rest of the text to justify such a hermeneutic move. *Rinrigaku* also makes due allowance for individuals resisting the unjustifiable demands of socio-ethical wholes: although it is committed to a non-transcendent understanding of ethics, it does not thereby fall into the trap of treating moral values as a function of societies’ contingent practices. It is precisely in the iterations of the movement of double-negation that individuals can access moral truth, and hence either non-heteronomously comply with socio-ethical demands, or resist them on the ground that, albeit socio-ethical, they are not genuinely ethical.

## Footnotes

1 Compare Fuller (1940, pp. 8–9), arguing, in the context of legal interpretation, that the objective of a statute is sometimes more apparent to a reader than its drafter; and Arendt (1982: 61–64) discussing Kant’s argument that authors and their genius is not prior to audiences and their taste, a faculty that enables them to discriminate between successful and unsuccessful performances of authorial genius.

2 Bellah (1965) offers an intertextual reading of *Rinrigaku* and some of Watsuji’s other ethical, as well as political, writings. Sevilla (2014) discusses how volumes two and three of *Rinrigaku* (still untranslated) develop and re-orient Watsuji’s ethical thinking as set out in the first volume.

3 Bellah (1965, p. 591) claims that even in its revised version, *Rinrigaku* and Watsuji’s other post-war writings ‘never’ find ‘another standard of value that can transcend the Emperor’.

4 At the same time, Watsuji makes clear that ‘individual and social modes of existence are abstractions from the dynamic and concrete reality of human beings in the world’ (Johnson 2016, p. 225). According to Higaki (2016, p. 461), for Watsuji the ‘isolated individual expresses nothing but a “privation” of the public’, ‘where the public nature of the self, the relationships that are already involved whenever the self discovers itself, is “abstracted away”’, leaving … an isolated subjectivity lacking all connection.’

5 This passage strikes us as self-refuting. It is unclear, specifically, that the point that ‘coercion constitutes the essence of obligation’ sits well with the point that ‘the negated individual obeys this authority out of respect’; indeed, if coercion is the essence of obligation, we would rather expect the individual to obey out of fear of sanctions.

6 It is not necessary for individuals to actually revolt and be forced back into association. It is enough to have the consciousness of revolt, so that coercion can also work as *the threat* of coercion, as is clear from Watsuji’s (1996, p. 115) discussion of the power of love in romantic relationships.

7 This is also clear from how Watsuji (1996, p. 110) approvingly discusses Durkheim, who ‘emphasized that society stands as the other to individual consciousness’, and who criticised the sociological ‘viewpoint that tries to deal with society, [rather than in terms of coercion,] as reciprocal mental activities’, a viewpoint whose tendency is to dissolve ‘individual consciousness … in society.’

8 Later, in discussing goodness and badness, Watsuji (1996, p. 284) argues that the revolt against wholeness is not actually bad if ‘the self and other opposed to each other in individualization, are already anticipating the unity of the future in and through this present opposition … [so that individualization is already] pierced through with the moment of coming back’. Similarly, then, when autonomous compliance with socio-ethical obligations might appear to dissolve the individual in the whole, we should instead recognise that compliance is pierced through with the possibility of future revolt, enabling consciousness of individuality to persist in compliance.

9 This part of Watsuji’s thought is rooted in Buddhism (Bellah 1965, p. 584; Lafleur 1978; Nagami 1981; Sevilla 2017).

10 At the same time the mutual negation of individuals and socio-ethical wholes gestures towards their common ground, for to ‘think of a thing is to distinguish it from something else, and yet in order to make such a distinction, the two must already have had something in common’ (Carter and McCarthy 2019). That common ground is precisely absolute negativity – the emptying of individuals and socio-ethical wholes into the Absolute, or ‘that totality beyond all limited, social totalities, namely the emptiness or nothingness at the bottom of all things’ (Carter and McCarthy 2019).

11 Recall that universal brotherhood is purely an ideal and an abstraction.

12 While Bernier (2006, p. 89) may be right in arguing that ‘instances of justified revolt are peripheral in [Watsuji’s] analysis’, it seems to us that we should not try to explain them away: doing so would not do justice to the text’s intention – or that is what this paper hopes to show.
